# Conquistar corpos, domar a selva: saúde e destruição na história do desenvolvimento da Amazônia

**DOI:** 10.1590/0102-311XPT263225

**Published:** 2026-05-01

**Authors:** André Luiz Machado das Neves

**Affiliations:** 1 Universidade do Estado do Amazonas, Manaus, Brasil.

O livro *Amazônia na Era do Desenvolvimento: Saúde, Políticas e Destruição, 1930-1966*, de Rômulo de Paula Andrade ^1^, laureado com o Prêmio ABEU (Ciências Sociais), constitui uma cara contribuição ao campo da Saúde Coletiva. A obra revisita um período decisivo em que a Amazônia foi convertida em fronteira estratégica do Estado brasileiro, especialmente entre o Estado Novo e o início da ditadura militar. Por meio de pesquisa documental em arquivos brasileiros e estadunidenses, Andrade demonstra de que modo saúde, nutrição e infraestrutura foram articuladas como técnicas de conquista do território e de governamento da vida, um projeto que, ao mesmo tempo em que prometia integração e progresso, produzia silenciamentos e destruições profundas.

O prefácio da obra destaca que a palavra “conquista”, presente reiteradamente no livro e utilizada como chave analítica para entender a imaginação política da Amazônia, é o que melhor expressa as ações na direção da Região Amazônica. O conceito justificava processos de intervenção sistemática que tinham como horizonte transformar corpos e paisagens para alinhá-los ao projeto desenvolvimentista brasileiro. Portanto, conquista, como o prefaciante aponta, nunca foi sinônimo de cuidado com a vida na história da Amazônia, rima mais com destruição do que com preservação ou conservação. Ao iluminar essa operação discursiva, o livro oferece ao campo da Saúde Coletiva ferramentas para historicizar determinantes estruturais das desigualdades sanitárias amazônicas.

Nesse sentido, se a saúde ocupou lugar central na estratégia estatal, o livro mostra como ela se tornou técnica de Estado. Desde o primeiro capítulo, o autor destaca a retórica sanitária que legitima o projeto de dominação no território, por meio do combate às doenças e o saneamento, que aparecem como condição prévia ao progresso. O segundo capítulo recupera a figura de Evandro Chagas, cuja atuação posiciona a ciência como instrumento de soberania e vigilância. O terceiro, ao tratar do Serviço Especial de Saúde Pública (SESP) e da cooperação Brasil-Estados Unidos durante a Segunda Guerra Mundial, revela uma dimensão frequentemente silenciada, isto é, a saúde como geopolítica. Controlar a malária, aqui, é controlar corpos e fronteiras, estruturando uma inserção internacional que articula higiene, ciência e militarização.

Essa dinâmica dialoga com Michel Foucault ^2^, para quem a saúde moderna se torna governo da vida, cálculo e administração de populações. Andrade demonstra que medir corpos, catalogar doenças ou reconfigurar dietas eram maneiras de governar. No quinto capítulo, a análise da Civilização da Mandioca parte da crítica de que a fome nunca foi destino natural, mas produto de políticas e omissões. A alimentação surge, então, como biopolítica do desenvolvimento, de forma que o Estado define o que come a população e, por consequência, quem ela pode ser.

A amplitude transnacional da conquista é outro mérito da obra. O livro destaca a atuação de instituições internacionais, que, junto ao Estado brasileiro, transformaram a Amazônia em laboratório global do desenvolvimento. A obra desmonta ficções que tomam a região como espaço atrasado para legitimar intervenção. A ajuda internacional mostra-se, assim, como política de poder, não de autonomia.

Observa-se, por sua vez, a invisibilidade dos próprios povos amazônicos. Em diálogo com reflexões, como as de Fraxe et al. ^3^, a Amazônia nunca foi vazia, tampouco homogênea; foi historicamente habitada por sujeitos híbridos, ribeirinhos, ameríndios, negros e migrantes que reinventaram modos de viver em territórios marcados por violência, sincretismo e sobrevivência criativa. A narrativa modernizadora, que convertia a floresta em laboratório e seus habitantes em paisagem, operou também como um regime de silenciamento, congelando identidades em imagens românticas ou atrasadas, ignorando a complexidade de seus mundos sociais.

O capítulo sobre a rodovia Belém-Brasília sintetiza as ambivalências do projeto modernizador. Ao recortar o território, a estrada anuncia a redenção e, simultaneamente, amplia vulnerabilidades, deslocamentos populacionais, desmatamento e conflitos socioambientais. Aqui, a noção de progresso se desnuda e não há integração sem dominação; não há desenvolvimento sem destruição. O livro oferece uma análise cuidadosa das tensões internas ao projeto desenvolvimentista, mostrando sua complexidade política.

O livro proporciona refletir a dimensão em que o desenvolvimento não apenas reorganizou ecossistemas, mas também inventou ausências, produziu categorias sociais e projetou sobre esses povos uma condição fantasmagórica entre a natureza e o atraso, entre o exótico e o descartável ^3^.

A obra contribui para a Saúde Coletiva ao revelar a genealogia de desigualdades que moldam a região até hoje. Mostra que a precarização da saúde, a reincidência de doenças, a insegurança alimentar e os impactos ambientais não são deficiências ou falhas, mas resultados históricos de escolhas políticas estruturais. Andrade ^1^ evidencia que a saúde brasileira se institui como dimensão política da vida social, inseparável de disputas sobre território e poder. Essa compreensão dialoga com abordagens mais contemporâneas sobre saúde planetária e justiça socioambiental, que ampliam radicalmente o escopo da Saúde Coletiva.

No contexto da COP30, em Belém (Pará), o livro ganha potência renovada. Ao recolocar a Amazônia no centro da agenda climática global, governos e corporações evocam a ideia de futuro sustentável do planeta. A obra convoca a lembrar que essa centralidade não é nova, pois a Amazônia sempre foi disputada como fronteira do progresso brasileiro, ontem com seringais, laboratórios e rodovias; hoje com mercados de carbono, megaprojetos verdes e retórica de transição energética. A história ensinada pelo livro ajuda a reconhecer continuidades inquietantes: Quem define o que é sustentável? Quem paga o preço da proteção ambiental? Quem decide quais vidas valem ser defendidas?

Ao historicizar o projeto desenvolvimentista, o livro nos mobiliza a confrontar uma verdade incômoda: não basta trocar o nome da conquista. Se, no século XX, a saúde abriu caminho para o domínio da selva, no XXI ela pode novamente ser convocada, agora em nome de salvar o clima, sem que se alterem as hierarquias políticas que estruturam a região. Nesse sentido, a obra opera um gesto ético e epistemológico, que é a recusa de naturalizar a desigualdade e reivindica a centralidade da Amazônia como local onde o futuro do mundo é produzido com e contra projetos hegemônicos. Desse modo, sem escutar os sujeitos amazônicos, o projeto de desenvolvimento torna-se também um projeto de apagamento ^3^.

A obra é, assim, uma leitura indispensável à Saúde Coletiva porque desmonta o mito da neutralidade técnica. Revela como diagnósticos, campanhas, dietas e estradas governam vidas, e como o território é produzido politicamente. As perguntas abertas pela obra são também as nossas. Que formas de cuidado podem emergir quando reconhecemos que existem mundos amazônicos para além da conquista? Em um momento em que a Amazônia volta a ser palco das disputas planetárias, o livro nos chama a imaginar políticas de vida que não passem pela destruição, com vistas a reinventar os percursos de uma história marcada pela desigualdade e inferiorização, imposta por um projeto civilizatório que tem como marca a domesticação das múltiplas alteridades amazônicas.
